# Case Report: Nystagmus as a core presenting sign in pediatric anti-Ma2 antibody-associated cerebellar ataxia: diagnostic implications from clinical and serological profiling

**DOI:** 10.3389/fped.2025.1659483

**Published:** 2025-10-15

**Authors:** Jiehui Ma, Yuting Yang, Zhiyu Li, Qianqian Tan, Pan Cao, Kun Ni, Xiaofang Cai, Dan Sun

**Affiliations:** ^1^ Department of Neurology, Wuhan Children’s Hospital of Tongji Medical College, Huazhong University of Science and Technology, Wuhan, China; ^2^ Emergency and Critical Care Medical Center, Wuhan Children’s Hospital of Tongji Medical College, Huazhong University of Science and Technology, Wuhan, China; ^3^ Department of Marketing, Wuhan Kindstar Clinical Diagnostic Institute Co., LTD, Wuhan, China; ^4^ Department of Research and Development, Mainuo (Wuhan) Medical Biotechnology Co., LTD, Wuhan, China

**Keywords:** Ma2, cerebellar ataxia, pediatric patient, cell-based assay, nystagmus

## Abstract

**Background:**

The anti-Ma2 antibody is a well-known, specific marker of paraneoplastic limbic and brainstem encephalitis, mainly described in adult, especially in males with testicular germ cell tumor. Pediatric cases remain exceptionally rare. We present a child with anti-Ma2 antibody-associated cerebellar ataxia in whom nystagmus was identified as a core presenting symptom; the diagnosis was confirmed via cell-based assay (CBA).

**Case presentation:**

An 11-year-old boy sought medical attention for symptoms such as vomiting, nystagmus, dizziness, slurred speech, and limb weakness. Routine laboratory tests and brain MRI were normal, simultaneously we ruled out infectious factors. Further limb coordination tests suggested the boy may have cerebellar ataxia. Based on clinical symptoms and the above tests, the boy underwent a comprehensive examination for suspected paraneoplastic neurological syndrome (PNS). Serum enzyme-linked immunospot test was positive (+) for anti-Ma2 antibodies and confirmed by CBA with a titer of 1:10. The boy was diagnosed with anti-Ma2 antibody-associated cerebellar ataxia. Subsequently, he received intravenous immunoglobulin (IVIG) and methylprednisolone (mPD) treatment and experienced significant symptomatic improvement. Complete resolution occurred by 38 days post-discharge, sustained through one-year follow-up.

**Conclusions:**

Nystagmus was first identified in pediatric patients with anti-Ma2 antibody-associated syndrome, expanding clinicians' knowledge of the phenotype in children. Our case demonstrates that IVIG and steroids induced rapid and sustained remission despite presumed cell-mediated immunity cases, with complete symptom resolution within 8 weeks and no recurrence at 1-year follow-up. We also emphasize CBA's superior accessibility and its higher sensitivity and specificity in detecting low-titer antibodies for detecting antibodies in autoimmune encephalitis, particularly in mild or atypical presentations.

## 1 Introduction

Anti-Ma2 antibody-associated neurological syndromes, including limbic encephalitis, diencephalic dysfunction, brainstem encephalitis, cerebellar ataxia, opsoclonus-myoclonus syndrome, and choreiform disorders, represent rare autoimmune conditions often linked to underlying malignancies. Intracellular Ma2 antigens mediated by T-cells are considered the primary pathophysiology of the disease (1). It has also been characterized as paraneoplastic neurological syndromes (PNSs) because it is associated with malignancies in more than 90% of cases (2, 3). However, the occurrence of anti-Ma2 antibodies in a child with neurological symptoms is extremely rare. The diagnosis of anti-Ma2 antibody-associated syndromes in children has been challenged by misleading clinical manifestations. Diagnosis and treatment of the disease, therefore, rely primarily on clinical experience from previously reported cases.

In order to facilitate its diagnosis, we reported a case of an 11-year-old boy who tested positive for anti-Ma2 antibodies and presented with cerebellar ataxia. A systematic literature review of previous cases was also conducted to gain a better understanding of the clinical manifestations, biological features, treatment, and outcomes of children with anti-Ma2 antibody-associated syndrome.

## 2 Materials and methods

This study received ethical approval from Wuhan children's hospital, Tongji medical college huazhong university of science & technology (Approval No. 2024R094-E01) with written informed consent obtained from the patient's guardian. An 11-year-old boy presenting with acute nystagmus, cerebellar ataxia, and vomiting underwent comprehensive evaluation including standardized neurological assessments [Medical Research Council (MRC) muscle grading, finger-to-nose test, heel-to-shin test]; serological/cerebrospinal fluid (CSF) analysis for infections and autoantibodies [anti-Ma2 antibodies confirmed via cell-based assay (CBA) with fluorescence colocalization criteria]; encephalomyocarditis virus (quantitative real-time polymerase chain reaction, qPCR), brain magnetic resonance imaging (MRI). Electrophysiological studies comprised nerve conduction studies (bilateral peroneal/tibial/median/ulnar nerves) and electrocardiogram (ECG). Initial and annual tumor surveillance including testicular ultrasound, whole-abdomen ultrasound, chest computed tomography (CT), and serum tumor markers [alpha-fetoprotein (AFP), beta human chorionic gonadotropin (β-HCG), carbohydrate antigen 199 (CA199) and cancer antigen 125 (CA125)].

The CBA assay required fulfillment of both technical and interpretative criteria for positivity. Technical validation: 1) examination of ≥3 independent microscopic fields; 2) ≥3–5 cells per field demonstrating complete co-localization of red (anti-Ma2 antibody) and green (GFP-tagged Ma2 antigen) fluorescence). For a positive result, every field examined must meet all the following criteria: 1) Whole-Cell colocalization: complete co-localization of red and green fluorescence throughout the cellular architecture (cytoplasm/membrane), excluding partial or marginal staining patterns; 2) Signal intensity: red fluorescence intensity in antigen-expressing cells significantly exceeds background staining in non-transfected cells; 3) Control validation: negative controls show no/background-level red fluorescence; 4) Titer confirmation following morphological assessment: positivity was first determined by cellular colocalization patterns, then confirmed by correlating signal intensity with antibody titer. Negative Criteria (any condition): 1) No red signal: only green fluorescence; 2) No colocalization: red fluorescence without green; 3) Control failure: green-fluorescent cells show non-specific scattered red fluorescence (background interference).

A systematic literature review was conducted using PubMed/Medline (2000–2025) with the keywords “pediatric anti-Ma2 encephalitis,” “anti-Ma2 cerebellar ataxia,” “nystagmus,” “paraneoplastic neurological syndromes," and “autoimmune cerebellar ataxia.” The analysis included all reported cases of anti-Ma2 antibody-positive children (<18 years) with neurological symptoms, with extracted data encompassing demographics and clinical features as summarized in Table 1.

**Table 1:** Summary of pediatric cases of anti-Ma2 antibody-associated neurological syndromes.

Case	Sex/Age (years)	Clinical presentation	Brain MRI scan	CFS	Associated tumor	Treatment	Outcomes	Reference
1	F/2	Decreased level of consciousness, speech and behavioral disorders	Occipital T2 signal enhancement	Normal	None	mPD	Partial recovery	Douma et al. (4)
2	M/2.5	Speech and behavioral disorders, swallowing difficulties, drooling	Increased T2 signal in right frontal areas	Normal	None	IVIG + mPD + Azathioprine	Partial recovery	
3	F/2	Speech and behavioral disorders	Normal	Normal	None	IVIG + mPD	Partial recovery	
4	F/8	Decreased level of consciousness, hallucination	External capsule Increased signal	Lymphocytic pleocytosis	None	IVIG + mPD + Azathioprine	Full recovery	
5	M/9	Confused mental state, decreased motor power	Increased T2 signal, leptomeningeal enhancement	Lymphocytic pleocytosis	None	IVIG + mPD + rituximab	Full recovery	Kim et al. (5)
6	M/8	Bilateral strabismus, seizures	Enlargement of left lateral ventricle	–	None	IVIG + mPD	Partial recovery	Hu et al. (6)
7	F/2	Refractory focal seizures, fever	Increased T2 signal in left frontoparietal areas	Normal	None	IVIG + mPD	Partial recovery	Mrabet et al. (7)
8	F/4	Myoclonus, chorea, ataxia	Normal	Normal	None	IVIG + mPD + Adrenocorticotropic hormone	Partial recovery	Boniel et al. (8)
9	M/11	Emesis, nystagmus, dizziness, ataxia and confusion	Normal	Normal	None	IVIG + mPD	Full recovery	Current study

F, female; M, male; MRI, magnetic resonance imaging; CSF, cerebrospinal fluid; IVIG, intravenous immunoglobin; mPD, methylprednisolone; -, denote unknown.

## 3 Results

An 11-year-old boy with a history of ventricular septal defect presented at the local clinic due to unexplained vomiting and headaches. Initially evaluated by a primary care physician, he was misdiagnosed with a cold. Despite receiving antibiotics treatment for four days, his condition worsened. Consequently, on day 10 after symptom onset, he was admitted to Wuhan children's hospital, Tongji medical college huazhong university of science & technology. On the initial evaluation, he presented with emesis, nystagmus, dizziness, and limb weakness. Neurological examination: the patient was alert. Cranial nerves: horizontal nystagmus was observed. Slurred speech was noted. Facial movements and sensation were symmetric. Blood gas analysis and whole blood cell counts were normal. The results of laboratory tests, including electrolytes, myocardial enzymes, IgE antibodies, coagulation function, anti-thyroglobulin antibodies, anti-GAD antibodies, and hepatorenal function were all within normal ranges. CSF routine analysis showed no abnormalities. Screening for encephalomyocarditis virus (cytomegalovirus, coxsackie virus and enterovirus) and bacteria did not reveal any specific infections. These viruses were screened as they are common causes of pediatric acute cerebellitis mimicking autoimmune encephalitis (4, 5). Brain MRI scans revealed normal findings. A CT scan of the chest showed increased vascular markings in the lower lungs, suggesting chronic bronchitis.

**Figure 1:**
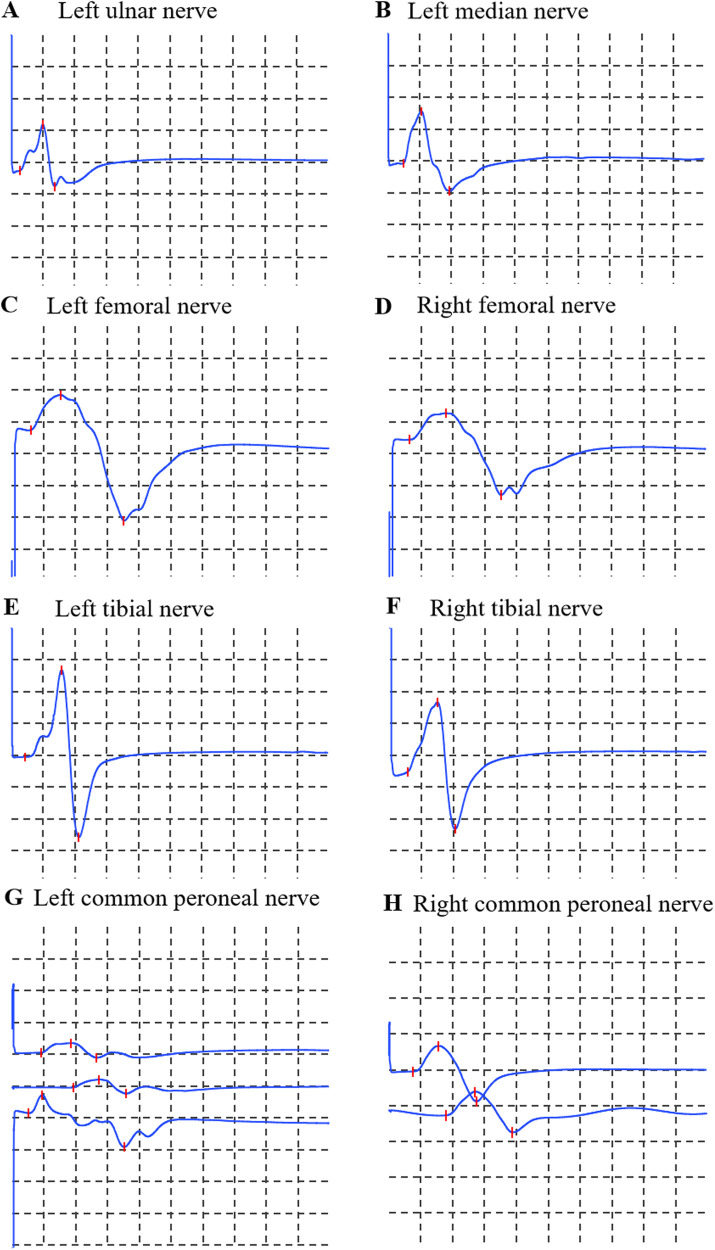
Motor nerve conduction studies. **(A)** Left ulnar nerve; **(B)** Left median nerve; **(C)** Left femoral nerve; **(D)** Right femoral nerve; **(E)** Left tibial nerve; **(F)** Right tibial nerve; **(G)** Left common peroneal nerve; **(H)** Right common peroneal nerve. Calibration: 5 mV/division (vertical), 5 ms/division (horizontal) for all traces.

Motor examination: muscle strength for foot dorsiflexion and all other muscle groups on the Medical Research Council scale were graded 5/5. Muscle tone was normal. Coordination: Finger-to-nose testing revealed dysmetria bilaterally. Heel-to-shin testing was impaired bilaterally. Sensation was intact to light touch and pinprick. Reflexes: Ankle jerks were 1+, while knee and upper limb reflexes were 2+ and symmetric. Plantar responses were flexor. Gait could not be fully assessed due to limb weakness and dizziness. Electrophysiological evaluation (nerve conduction studies) of the motor pathways revealed that the amplitude of the compound muscle action potential in the left peroneal nerve was within the normal range but lower than the right side. Other examined motor nerves (left median, ulnar, right peroneal, bilateral tibial, femoral) showed no abnormalities. Sensory nerve conduction studies (left median, ulnar, bilateral sural, superficial peroneal) and F-wave studies (left median, ulnar, bilateral tibial) were normal. Needle electromyography (EMG) of muscles innervated by the left ulnar, left median, bilateral femoral, tibial, and common peroneal nerves (including bilateral tibialis anterior) demonstrated no abnormal spontaneous activity, normal motor unit potentials, and full recruitment patterns, with the exception of asymmetric amplitude noted in the left common peroneal nerve (Figure 1). In addition, the electrocardiographic findings suggested that the patient had sinus bradycardia accompanied by arrhythmia. The electrocardiographic abnormality may be related to the history of ventricular septal defect operation.

**Figure 2:**
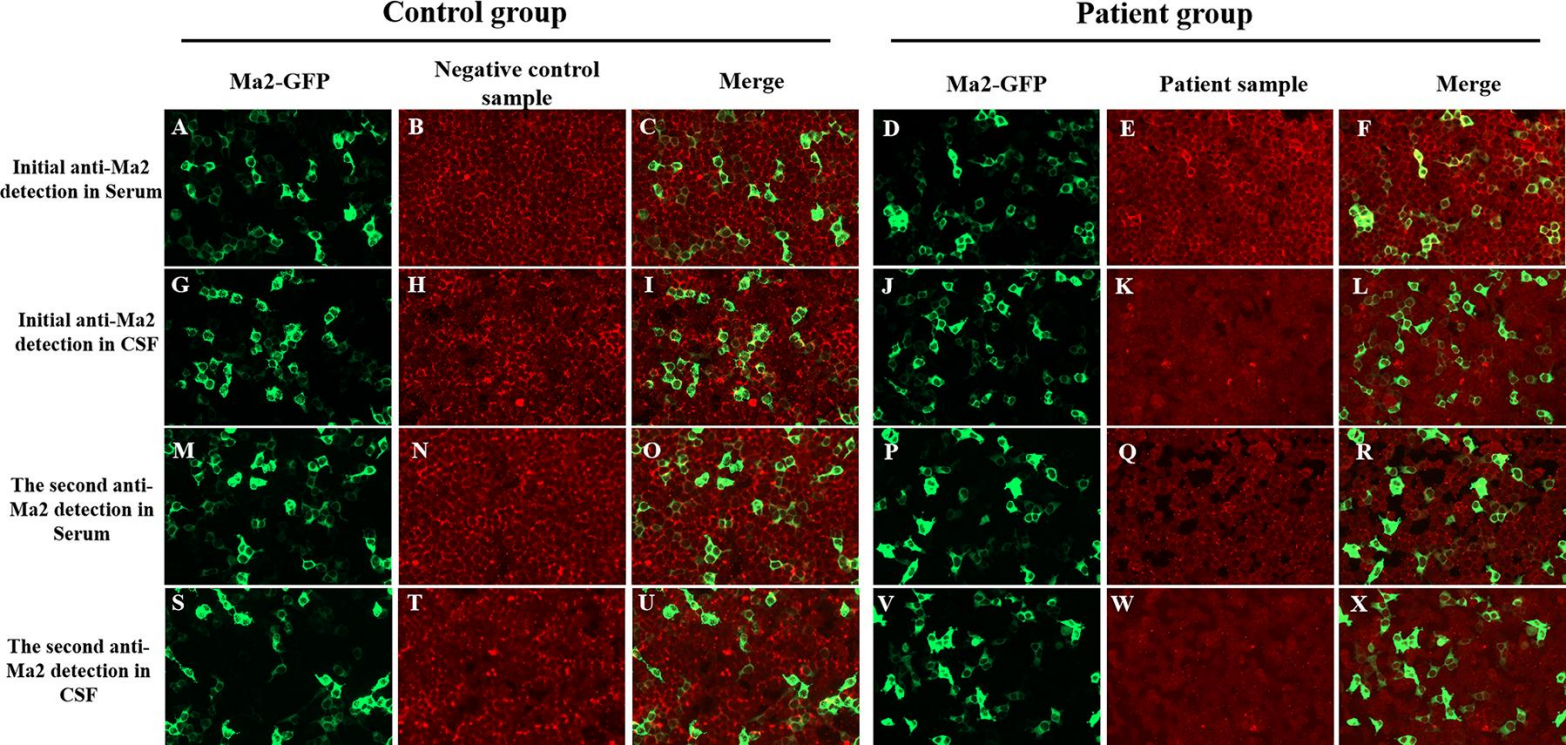
CBA results by fluorescence microscopy. (Left column of each group) HEK293 cells transfected with Ma2-GFP (green; panels **A,D,G,J,M,P,S,V**); (Middle column of each group) Anti-Ma2 antibody detection in serum/CSF (red; panels **B,E,H,K,N,Q,T,W**); (Right column of each group) Merged green Ma2-GFP and red anti-Ma2 fluorescence signals (panels **C,F,I,L,O,R,U,X**). In patient samples, initial serum testing **(E)** revealed low-positive anti-Ma2 Abs with specific colocalization in merge **(F)**, while all other samples (serum Q; CSF K, W) were negative for anti-Ma2, and their merged fluorescence signals **(L,R,X)** showed no colocalization. Control samples showed no anti-Ma2 reactivity in serum **(B,N)** or CSF **(H,T)**, with merged fluorescence signals **(C,I,O,U)** confirming absence of colocalization. (200× magnification). CBA, cell-based assay; Abs, antibodies; CSF, cerebrospinal fluid; HEK293, human embryonic kidney cells 293; GFP, green fluorescent protein.

Given this information, the patient underwent a comprehensive workup for suspected PNS due to the subacute onset of multiple neurological symptoms (ataxia, nystagmus, and slurred speech) that were not explained by infection or other metabolic causes. An Enzyme-linked immunospot assay performed on serum collected on admission day was positive (+) for anti-Ma2 antibodies. In order to confirm specificity and antibody titers, a CBA was performed and detected a weakly positive antibody in the serum at a dilution of 1:10 (Figure 2), confirming the diagnosis. The CBA was performed on the second day of hospitalization (symptom day 12), immediately prior to initiating IVIG therapy. With the discovery of weakly positive anti-Ma2 antibodies, the patient had a 3-day course of intravenous immunoglobulin (IVIG) at 400 mg/kg/day followed by mPD at 2 mg/kg/day for a week (oral taper was chosen instead of pulse steroids due to mild presentation and concern for cardiac side effects given his bradycardia).

**Figure 3:**
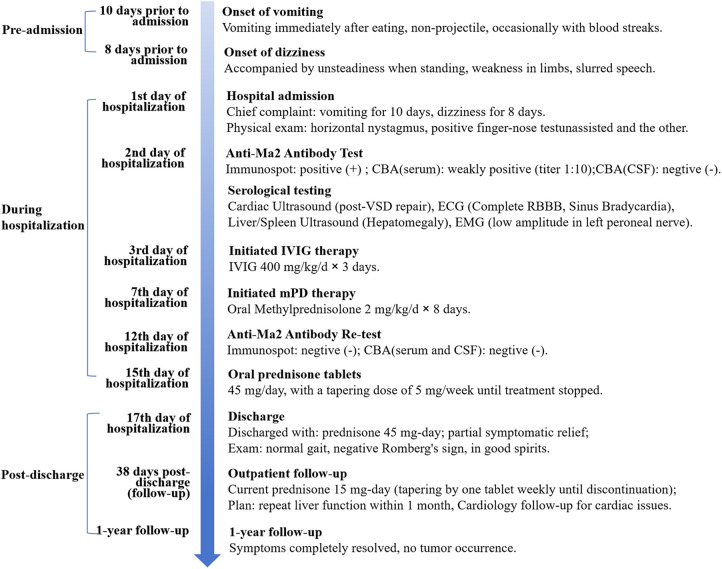
Clinical timeline of an 11-year-old male with anti-Ma2 antibody-associated cerebellar ataxia. Anti-Ma2, Anti-Ma2 antibody; CBA, cell-based assay; CSF, cerebrospinal fluid; ECG, electrocardiogram; EMG, electromyography; IVIG, intravenous immunoglobulin; mPD, methylprednisolone; qd, quaque die; RBBB, Right Bundle Branch Block; VSD, Ventricular Septal Defect. 1st: first; 2nd: second; 3rd: third; 7th: seventh; 12th: twelfth; 15th: fifteenth; 17th: seventeenth.

Repeat anti-Ma2 antibody test (Enzyme-linked immunospot) on second day of hospitalization was negative (-). Consistent with this, a second CBA conducted on the 5th day of mPD treatment showed both serum and CSF tests were negative (Figure 2). After intravenous treatment, oral mPD was given at 45 mg/day, with a tapering dose of 5 mg/week until the treatment was stopped. For bradycardia, atropine sulfate is chosen for treatment. The symptoms of headache and vomiting were significantly alleviated during hospitalization. At discharge (seventeenth day of hospitalization), neurological examination confirmed complete resolution of dizziness, nystagmus, and vomiting, with preserved consciousness, normal speech function, and intact limb motor strength/tone. Residual cerebellar impairment manifested as action tremor during object manipulation, slow gait with observable truncal sway, inability to maintain linear trajectory during ambulation, and upper limb dysmetria on finger-nose testing. During the 38-day post-discharge follow-up, complete resolution of all neurological symptoms was confirmed. No tumor occurrence was detected during subsequent annual imaging surveillance. The chronological progression of diagnosis, treatment response, and follow-up outcomes are detailed in Figure 3.

## 4 Discussion

Increased clinical awareness and improved identification of specific antibodies have been major advancement in the field of neurology. Ma2 is an intracellular onconeural protein selectively expressed by normal brain tissue and testicular tumors in patients. It was initially discovered by Voltz for diagnosing paraneoplastic syndrome and indicating testicular tumor (6). The mechanism underlying neuronal damage may involve inflammation mediated by cytotoxic T cells (7), although it is not completely understood.

Anti-Ma2 antibodies are more commonly identified in adults than in children. To the best of our knowledge, only nine pediatric cases associated with anti-Ma2 and neurological symptoms have been reported so far (Table 1). These cases involve four boys and five girls aged between two and eleven years old (Table 1). Major symptoms included mental status changes such as decreased consciousness (22.2%, 2/9) (8), movement disorders such as chorea (11.1%, 1/9) and myoclonus (11.1%, 1/9) (9), and speech dysfunction (33.3%, 3/9). These were often accompanied by seizures (22.2%, 2/9) or ataxia (22.2%, 2/9) (10, 11). While cerebellar ataxia is increasingly recognized as a key manifestation of anti-Ma2 syndromes (1, 3, 6), adult studies highlight its association with complex eye movement disorders—such as opsoclonus in oropharyngeal carcinoma (12) and multidirectional nystagmus in Hodgkin lymphoma (13)—whereas pediatric-specific data remain limited. In reported pediatric cases, eye movement abnormalities including opsoclonus-myoclonus and saccadic dysmetria have been reported (7–9, 11), but nystagmus as a core presenting sign has not been previously emphasized. Here, we report the first detailed description of nystagmus as a predominant symptom in pediatric anti-Ma2 antibody-associated cerebellar ataxia, expanding its clinical spectrum and underscoring its diagnostic significance across age groups.

Abnormal brain MRI findings were described in six of the nine cases, with the most common being an increased T2 FLAIR signal (8, 11, 14), which was seen in four patients. However, this signal was absent in our patient. The diagnosis of anti-Ma2 antibody-associated cerebellar ataxia in our patient was supported by the characteristic neurological presentation of cerebellar ataxia accompanied by brainstem involvement (nystagmus and slurred speech), demonstrating a pattern frequently associated with anti-Ma2 antibodies in both adult and pediatric populations (6). Although classical anti-Ma2 syndromes often involves limbic structures, our case aligns with the cerebellar-predominant variant reported in a subset of patients (10). While classic limbic dysfunction was absent, the subacute progression over two weeks contrasted sharply with the hyperacute onset typical of post-infectious ataxia. Comprehensive exclusion of alternative etiologies - including normal inflammatory markers (CSF WBC <5/μl, CRP <1 mg/dl), negative viral PCR panels for common pathogens, and absence of metabolic abnormalities (e.g., normal electrolytes, hepatic/renal function, anti-thyroglobulin, and anti-GAD antibodies) - further strengthened the diagnosis. The normal MRI findings, consistent with approximately 40% of anti-Ma2 antibody-associated cerebellar ataxia cases (10), underscore that radiographic absence does not preclude this diagnosis when supported by compelling clinical and serological evidence.

Regarding treatment, while anti-Ma2 antibody-associated disease is considered primarily T-cell mediated, our case and literature review show that IVIG can still be effective when combined with steroids. This may be due to IVIG's dual immunomodulatory effects: beyond neutralizing autoantibodies, it suppresses pathogenic T-cell activation via Fcγ receptor-mediated signaling and dendritic cell modulation (15). Although histopathology was unavailable, the prompt antibody clearance and symptom resolution suggest T-cell exhaustion following immunotherapy, as reported in seropositive paraneoplastic syndromes (16). The optimal treatment regimen remains unclear, especially in pediatric cases. In our case, we used lower-dose IVIG (400 mg/kg/d) for three days and oral steroids instead of pulse steroids due to the mild presentation and cardiac concerns, with good response. More aggressive therapies like rituximab or adalimumab could be considered for refractory cases (7).

Anti-Ma2 antibodies have traditionally been detected by Western blot analysis or immunospot in most previous studies. While CBA for the detection of anti-Ma2 antibodies has been reported, it remains relatively uncommon in the literature. In this study, we applied CBA for the detection of anti-Ma2 antibodies following an initial positive result by immunospot. CBA, which uses eukaryotic cell lines transfected with plasmids encoding the human sequence of the protein of interest, has higher sensitivity and specificity than traditional methods (17, 18). This two-step approach (immunospot followed by confirmatory CBA) significantly reduces the risk of false positives, the characteristic clinical triad, the prompt and significant clinical response to immunotherapy, and crucially, the correlation between antibody clearance (seroconversion within 5 days of steroid initiation) and clinical improvement strongly support the pathogenic relevance of the antibody in our case. The rapid seroconversion is likely a direct immunological effect of the immunotherapy (e.g., rapid suppression of antibody-producing cells or Fc receptor blockade) and aligns with the swift clinical recovery. This aligns with recent reports of clinically significant low-titer anti-Ma2 antibodies in pediatric encephalitis (14, 19). The combination of our stringent two-step confirmatory assay (immunospot followed by CBA) and the compelling clinical-immunological correlation—characteristic triad, rapid immunotherapy response, and crucially, synchronous antibody clearance (seroconversion within 5 days) with clinical improvement—provides robust evidence for the pathogenic role of anti-Ma2 antibodies and effectively rules out a false positive result. Due to the advantages of CBA in accurate diagnosis and differentiation of atypical cases, we recommend CBA as the preferred method for antibody detection in autoimmune encephalitis. There is no evidence of an underlying paraneoplastic tumor observed in our case, as well as in the other eight pediatric cases. Nevertheless, annual tumor screening every 5 years is still recommended for these pediatric patients due to the high risk of tumorigenesis in adult patients with anti-Ma2 encephalitis (19, 20).

First-line treatment with intravenous immunoglobulin or mPD was administrated to all patients. A second-line immunotherapy, including azathioprine and rituximab, was administrated to three patients. Although there is currently no evidence-based treatment for childhood autoimmune encephalitis, all nine patients achieved partial or even full recovery, suggesting that immunomodulatory treatments are the preferred option for immunosuppression over other treatments.

## 5 Conclusion

Overall, we documented an atypical case of anti-Ma2 antibody-associated cerebellar ataxia in an 11-year-old patient. Nystagmus was reported as a core presenting feature for the first time in this pediatric case, broadening our understanding of the characteristics of anti-Ma2 associated paraneoplastic syndromes. While anti-Ma2 antibody-associated disease is considered primarily T-cell mediated, our case demonstrates that IVIG combined with steroids can achieve rapid and sustained remission even in presumed cell-mediated immunity cases. The patient showed a favorable response to treatment with mPD and IVIG, achieving complete symptom resolution by 38 days post-discharge and sustained functional recovery with no recurrence at the 1-year follow-up. Furthermore, this report highlights CBA's superior accessibility and its higher sensitivity and specificity in detecting low-titer antibodies for detecting antibodies in autoimmune encephalitis, particularly in mild or atypical presentations.

## Data Availability

The datasets supporting the findings of this study are all available within the article (Table 1 and Figures 1–3). Further inquiries can be directed to the corresponding authors (DS).
